# Breast Cancer Surrogate Subtype Classification Using Pretreatment Multi-Phase Dynamic Contrast-Enhanced Magnetic Resonance Imaging Radiomics: A Retrospective Single-Center Study

**DOI:** 10.3390/jpm13071150

**Published:** 2023-07-18

**Authors:** Lucija Kovačević, Andrija Štajduhar, Karlo Stemberger, Lea Korša, Zlatko Marušić, Maja Prutki

**Affiliations:** 1 Clinical Department of Diagnostic and Interventional Radiology, University Hospital Centre Zagreb, Kispaticeva 12, 10000 Zagreb, Croatia; lucija.kovacevic1@kbc-zagreb.hr (L.K.); maja.prutki@mef.hr (M.P.); 2 Department for Medical Statistics, Epidemiology and Medical Informatics School of Medicine, University of Zagreb, Salata 12, 10000 Zagreb, Croatia; 3 Clinical Department of Pathology and Cytology, University Hospital Centre Zagreb, Kispaticeva 12, 10000 Zagreb, Croatia; 4 School of Medicine, University of Zagreb, Salata 3, 10000 Zagreb, Croatia

**Keywords:** radiomics, breast cancer biomarkers, precision medicine, machine learning, magnetic resonance imaging

## Abstract

This study aimed to explore the potential of multi-phase dynamic contrast-enhanced magnetic resonance imaging (DCE-MRI) radiomics for classifying breast cancer surrogate subtypes. This retrospective study analyzed 360 breast cancers from 319 patients who underwent pretreatment DCE-MRI between January 2015 and January 2019. The cohort consisted of 33 triple-negative, 26 human epidermal growth factor receptor 2 (HER2)-positive, 109 luminal A-like, 144 luminal B-like HER2-negative, and 48 luminal B-like HER2-positive lesions. A total of 1781 radiomic features were extracted from manually segmented breast cancers in each DCE-MRI sequence. The model was internally validated and selected using ten times repeated five-fold cross-validation on the primary cohort, with further evaluation using a validation cohort. The most successful models were logistic regression models applied to the third post-contrast subtraction images. These models exhibited the highest area under the curve (AUC) for discriminating between luminal A like vs. others (AUC: 0.78), luminal B-like HER2 negative vs. others (AUC: 0.57), luminal B-like HER2 positive vs. others (AUC: 0.60), HER2 positive vs. others (AUC: 0.81), and triple negative vs. others (AUC: 0.83). In conclusion, the radiomic features extracted from multi-phase DCE-MRI are promising for discriminating between breast cancer subtypes. The best-performing models relied on tissue changes observed during the mid-stage of the imaging process.

## 1. Introduction

Breast cancer is the most commonly diagnosed cancer worldwide [1]. Since 1989, breast cancer mortality rates have decreased by approximately 40%, mainly due to earlier diagnoses and treatment advances [2]. However, despite the decline in mortality in the past few decades, breast cancer is still the leading cause of cancer mortality in the female population, which can partly be attributed to tumor heterogeneity [1,3]. Both intertumoral and intratumoral heterogeneity represent diagnostic and therapeutic challenges resulting in unpredictable clinical outcomes and responses to existing therapy [3,4]. Therefore, the personalization of breast cancer care is crucial to improve treatment outcomes. Biomarkers are essential to enable a personalized approach to breast cancer patients. Nowadays, the most frequently used biomarkers used to guide diagnostic and treatment decisions for breast cancer patients are hormone receptors (estrogen, progesterone, and HER2 receptor) and the proliferation marker Ki-67 assessed by immunohistochemical analysis. Together, they define different surrogate subtypes of breast cancer [5]. The surrogate subtype and stage of the disease have a central role in optimal treatment selection for breast cancer patients. In treatment-naïve breast cancers, biomarkers that define surrogate subtypes can be assessed only through biopsy samples. However, the expression of these biomarkers can be highly variable within an individual tumor, making biopsy samples unrepresentative of the whole tumor [6]. Therefore, to provide personalized care for breast cancer patients, there is a need for an improved and more reliable classification of breast cancer surrogate subtypes that would be representative of the whole tumor and preferably less invasive and more affordable than biopsies.

Medical imaging is an affordable, routinely used, non-invasive method that captures the intratumoral heterogeneity and has, through radiomics, massive potential for developing a reliable classification of surrogate subtypes. Radiomics, a quantitative approach to medical imaging, is a bridge between medical imaging and personalized medicine aimed at enhancing the existing information available to clinicians [7,8,9]. It allows for a high-throughput extraction and analysis of many quantitative imaging features to improve diagnostic, prognostic, and predictive accuracy [9]. Radiomics may be applied to medical images from different imaging modalities.

Classical radiomics experienced rapid development starting in 2012. The typical classical radiomics workflow involves several distinct steps: (1) image acquisition, (2) segmentation of the region or volume of interest, (3) quantitative image feature extraction from the identified region, (4) quantitative image feature selection, (5) model development, and (6) evaluation of model performance [7,10]. Classical radiomics results in the development of machine-learning-based models, which have demonstrated high accuracy across various tasks, highlighting the potential of radiomics in clinical decision making. Nevertheless, classical radiomic models have faced challenges in terms of reproducibility due to the impact of various technical factors on the extracted radiomic features [8]. This limitation has hindered the widespread application of classical radiomics in clinical practice.

Recently, a novel approach called “deep radiomics” has been introduced, utilizing deep learning methods for model development. Deep radiomics holds great potential in addressing the limitations of conventional radiomics since deep networks can directly learn from images, reducing the number of steps that can be interfered with within the pipeline [11]. As expected, deep learning radiomics models have also shown impressive accuracy across different tasks.

In most studies, as reviewed by Demircioğlu, deep learning models may show better performance compared to classical machine learning models [11]. However, it is important to note that, in over 25% of the studies, deep learning models did not outperform classical machine learning models [11]. This suggests the need for additional investigation and comparison of both modeling strategies across diverse clinical applications.

The prediction of breast cancer subtype is a subject that requires extensive investigation due to its significant potential in determining the most effective treatment. Regarding breast cancer subtype classification radiomics, most published studies analyzed quantitative features using dynamic contrast-enhanced breast magnetic resonance imaging (DCE-MRI) [12,13,14,15,16,17,18,19,20,21,22,23,24,25,26]. While radiomics, relying on classical machine learning models such as gradient boosting methods, has been the primary approach in the majority of these studies [12,13,14,15,16,17,18,19,20], there is a growing body of research describing models based on deep learning [21,22,23,24] or both classical machine learning and deep learning techniques [25,26]. However, most of them used features extracted from single-phase DCE-MRI and reflected only the spatial heterogeneity at that time.

Although inconclusive results from previous studies have been reported regarding the kinetic curve pattern of different breast cancer surrogate subtypes, Kazama et al. revealed significant differences in the time–intensity curve patterns and receptor status [27,28,29]. Furthermore, different breast cancer surrogate subtypes exhibit different angiogenic properties [30], which may result in different enhancement patterns during scanning.

In terms of radiomics, it has been shown that subtype classification models that use multi-phase DCE-MRI data were generally better than models using single-phase DCE-MRI data [26]. In their study employing the classical radiomics methodology, Xie et al. reported that texture features extracted from original images of DCE (dynamic contrast-enhanced) and DWI (diffusion-weighted imaging), as well as those changing over six time points or three b-values, achieved the highest accuracy of 72.4% for classifying four breast cancer subtypes [18]. Sun et al. developed a prediction model based on multi-phase DCE-MRI utilizing a combination of convolutional neural networks (CNNs) and machine learning techniques to distinguish between luminal and non-luminal breast cancer subtypes. Their model achieved an impressive area under the receiver operating characteristic curve (AUROC) ranging from 0.867 to 0.958 on the testing dataset [25]. Zhang et al. employed deep learning algorithms, including a conventional convolutional neural network (CNN) and a recurrent CNN, to differentiate three breast cancer subtypes using multi-phase DCE-MRI [23]. The models achieved an accuracy of 0.8–0.9 during training, but it was observed that the developed model could not be directly applied to an independent testing dataset acquired from a different hospital using a different scanner [23]. The findings from the observed studies indicated that employing both classical and deep radiomics approaches based on multi-phase DCE-MRI could lead to an improved classification of surrogate subtypes. However, an important limitation identified in these studies was the small sample size, which affected the study design by not including all surrogate subtypes separately, despite their distinct treatment strategies.

Regarding model and feature interpretability, it is important to highlight that classical radiomic features are generally considered to be more interpretable by radiologists in comparison to deep features, despite the advantages offered by deep radiomics [31]. However, it is worth noting that the interpretability of a model is rarely emphasized in radiomics studies, which can impact its acceptance and comprehension among radiologists.

This study aims to examine the potential of multi-phase DCE-MRI radiomic signatures for surrogate subtype classification in breast cancer patients by extending the range and type of images being analyzed, as well as offering an interpretation framework for a thorough analysis of the developed models, features, and image types that the models rely on. Thus, here, we investigate the utility of radiomic features from the multi-phase DCE-MRI in enabling better surrogate subtype classification.

## 2. Materials and Methods

### 2.1. Patients and Study Design

This single-center retrospective study was approved by the institutional review board, and the requirement for informed consent was waived. The hospital information system was reviewed for patients who underwent core-needle biopsy between January 2015 and January 2019. The inclusion criteria were that the patient had a core-biopsy-confirmed invasive breast cancer with a surrogate subtype determined on core-needle biopsy sample or surgical specimens before any systemic or other locoregional treatment and that the patient underwent breast DCE-MRI imaging at the UHC Zagreb before any treatment. The exclusion criteria were as follows: (a) prior and concurrent malignancies, (b) earlier breast surgery, (c) inflammatory breast cancer, and (d) poor image quality. Finally, 319 patients were enrolled in this study.

### 2.2. Histopathological Analysis

Histopathological reports were retrospectively reviewed from the hospital information system. The histopathological report of both core-needle biopsy samples and surgical specimens included assessment of the histological type (according to the *WHO Classification of Tumours of the Breast*, Fourth Edition) and the histological grade (according to Nottingham histological grade), and immunohistochemical (IHC) analysis of the estrogen receptor (ER), progesterone receptor (PR), HER2, and Ki-67 status. The status of the ER, PR, and HER2 receptors was assessed according to the joint American Society of Clinical Oncology (ASCO)/College of American Pathologist (CAP) guidelines for BC testing. The ER or PR status was considered positive if ≥1% of tumor cells demonstrated positive nuclear staining by immunohistochemistry. The HER2 status was determined positive when the IHC staining intensity score was greater than or equal to three or when the IHC staining intensity score was equivalent to two and the HER2/CEP17 ratio was ≥2.0 and the average HER2 copy number was ≥4.0 as determined by silver in situ hybridization (SISH) according to the American Society of Clinical Oncology (ASCO)/College of American Pathologists (CAP) guideline recommendations for HER2 testing in breast cancer. Surrogate definitions [5] based on IHC analysis of breast cancer tissue were used, and the subtypes were determined based on the receptor status as luminal A like (ER+, PR+ (≥20%), HER2-, Ki67 < 20%); luminal B-like HER2 positive (ER+ and/or PR + (<20%), HER2+, Ki67 > 20%); luminal B-like HER2 negative (ER+ and/or PR+, HER2-, Ki67 > 20%); HER2 positive (ER-, PR-, HER2+); and triple negative (ER-, PR-, HER2-).

### 2.3. Dynamic Contrast-Enhanced Magnetic Resonance Imaging Acquisition Protocol

MR images were acquired between January 2015 and January 2019 with a 1.5 T MR unit (Avanto, Siemens, Erlangen, Germany) using a dedicated breast coil with the patient in a prone position. For the purposes of this study, only the 3D T1-weighted FatSat axial sequence (TR = 4.06, TE = 1.65, FA = 10.0, matrix 1.0 × 0.8 × 1.5, thickness 1.50, interval 20.0%, FOV = 320 mm, NEX = 1) acquired before and five times after intravenous administration of 0.1 mmol/kg body weight of contrast agent gadoterate meglumine (Dotarem^®^, Guerbet, Princeton, NJ, USA) was used. Contrast material was injected into the antecubital vein with a 20 G needle at a flow rate of 3.5 mL/s, followed by a flush of 15 mL of saline solution. The post-processing evaluation of all breast MRI exams included image subtraction of the dynamic images and maximum-intensity projections (MIPs) of subtracted data to better identify enhancing lesions.

### 2.4. Image Segmentation

A breast imaging expert with over 15 years of experience manually segmented all breast cancers presenting as a mass with enhancement on the DCE-MRI of the breast. Lesion segmentation was performed in three dimensions (3Ds), and the regions of interest (ROIs) were delineated manually using the ITK-SNAP software (version 3.8.0.) [32] on each slice of the first post-contrast T1W sequence by excluding marker artifacts. The first post-contrast T1W sequence was used as it is considered the peak of enhancement of breast cancers.

### 2.5. Radiomic Feature Extraction, Selection, and Modeling

Radiomic feature extraction from the segmented ROIs was performed using the open-source Pyradiomics [33] library and self-written scripts in Python 3.8 programming language. A total of 1781 IBSI-compliant 3D volumetric radiomic features from manually segmented breast cancers were extracted from the native and five post-contrast DCE-MRI sequences as well as from the subtraction images. The extracted features were also considered in the temporal dimension, given the period elapsed from the native to the last (fifth) post-contrast sequence. From the extracted features for each sequence, we combined all values of the feature through all sequences and developed the mean, standard deviation, minimum, maximum, covariance, and maximum difference for each patient, as well as a set of concatenated features from all sequences. The extracted radiomic features comprised 14 shape features, 18 first-order intensity statistics features, and 75 texture features including 24 from the gray-level cooccurrence matrix (GLCM), 14 from the gray-level dependence matrix (GLDM), 16 from the gray-level run length matrix (GLRM), 16 from the gray-level size zone matrix (GLSZM), and 5 from the neighborhood gray-tone difference matrix (NGTDM). All were extracted from both the original volumes (107 features) and the subtracted volumes, without any pre-processing prior to the radiomics pipeline, which extracts features from both original images and images with 18 applied filtering procedures (in total, 18 × 93 features) [33]. The filtering procedures used five simple filters including square, square root, logarithm, exponential, and gradient magnitude of the volume intensities. In addition to these simple filters, we also use wavelet filtering with all the possible combinations of applying either a high- or a low-pass filter in each of the three dimensions (i.e., in total, eight decompositions, oct-bands), as well as a Laplacian of Gaussian (LoG) filter with five different widths (2, 3, 4, 5, and 7 mm) of the filter in the Gaussian kernel (i.e., so-called edge enhancement filter with different levels of coarseness). Finally, we obtained, in total, 8 × 93 wavelet features and 5 × 93 LoG features. As the 14 shape features are independent from intensity values, they were extracted only for the original (unfiltered) volumes.

In the feature selection process, a univariate analysis was performed first, using the Mann–Whitney U test to compare radiomic features between surrogate subtypes. All features were ranked according to the *p*-value from the statistical test in ascending order, and the top 5% of features were used for further analysis. Second, the Pearson correlation coefficient between each pair of features was computed (denoted as r hereafter). All pairs of features with |r| > 0.85 were detected, and the feature in each of these pairs with the larger P-value from the Mann–Whitney U test was removed from the feature set. Finally, a random-forest-based feature selection method named Boruta [34] was used to detect the key features for surrogate subtype classification. The details and a list of features selected for each sequence can be found in the table in the Supplementary Material.

The model development involved logistic regression, classification and regression trees (CARTs), support vector machines (SVMs), and two prevalent tree-based ensemble methods—random forest and gradient-boosting trees. The primary cohort, consisting of 288 segmented breast cancers, was subjected to ten repeated rounds of five-fold cross-validation (CV) to internally validate and select the model. Each round of CV generated five unique splits of the primary cohort into training and validation sets, ensuring that every data point appeared in a validation set exactly once in each round. The performances of the models were then evaluated on an independent validation cohort, which comprised 72 cancer segmentations.

Table 1 and Table 2 present the results from the independent validation set for different sequences and different models. This evaluation was performed using models with the best CV score from the primary cohort. The performance of these models was quantified using the area under the receiver operating characteristic curve (AUROC) and area under the precision–recall curve (AUPRC). The overall AUC was computed using micro-averaging, attributing equal importance to each example. By focusing on the micro-average, we ensure that the highest importance is given to the most prevalent categories.

For multiple classification tasks, we applied stratified random sampling to ensure that each class was proportionally represented in both training and validation sets during each round of CV. This helped maintain the balance of data across all tasks.

### 2.6. Model Interpretability

Shapley additive explanations (SHAPs) is a method for interpreting complex machine learning models and was used to enhance the interpretability and comprehension of the radiomic features obtained from the radiomics model. SHAPs is a game-theoretic approach commonly employed to explain the output of a tree-based machine learning model. It enables the identification of the contribution of individual features to a model’s prediction. The SHAPs method was used to explain the predictions of the logistic model that classified breast carcinomas into one of five different subtypes.

## 3. Results

### 3.1. Breast Cancer Characteristics

A total of 360 breast cancers from 319 Caucasian female breast cancer patients were included in this study. The mean age was 56 years (range: 28–85 years). The cancers were classified into five classes according to surrogate subtype, including triple-negative, 33 (9.17%); human epidermal growth factor receptor 2 (HER2)-positive, 26 (7.22%); luminal A-like, 109 (30.28%); luminal B-like HER2-negative, 144 (40.0%); and luminal B-like HER2-positive, 48 (13.33%), breast cancers. The predominant histological subtype was invasive carcinoma of no special type, 313 (86.94%), followed by invasive lobular carcinoma, 36 (10%); invasive mucinous carcinoma, 4 (1.11%); invasive tubular carcinoma, 4 (1.11%); invasive micropapillary carcinoma, 2 (0.56%); and invasive papillary carcinoma, 1 (0.28%).

### 3.2. Predictive Modeling

**Figure 1:**
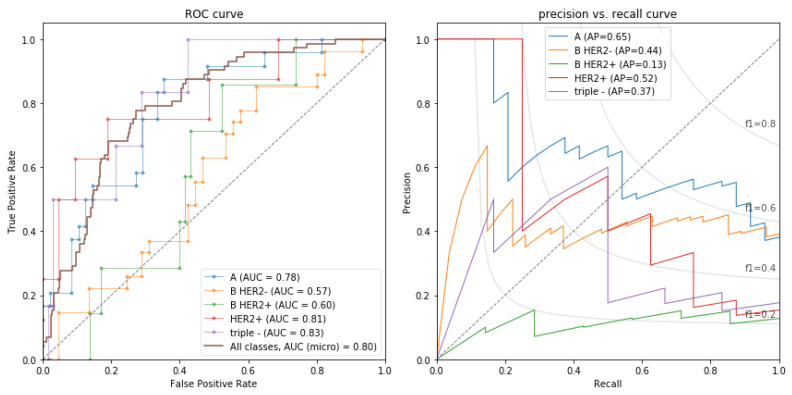
The best-performing model was logistic regression trained and yielded an area under the receiver operating characteristic curve (AUC) of 0.80 in both primary and independent validation cohorts. Shown here are the per-class and overall ROC (**left**) and precision–recall curves (**right**) of the best model. Abbreviations for different surrogate subtypes are as follows: A—luminal A like; B HER2−—luminal B-like HER2 negative; B HER2+—luminal B-like HER2 positive; HER2+—HER2 positive; triple−—triple negative.

All the models were optimized using the Bayesian optimization method implemented in the Python scikit-optimize library. Although several models performed similarly, the best-performing model was logistic regression trained and yielded an area under the receiver operating characteristic curve (AUC) of 0.80 in both primary and independent validation cohorts. Figure 1 shows the AUC and area under the precision–recall curve (AUPRC) of the best model for each surrogate subtype.

IBSI-compliant 3D volumetric radiomic features from manually segmented breast cancers were extracted from the native and five post-contrast DCE-MRI sequences as well as from the subtraction images. Several machine learning models were trained and optimized on each MRI sequence. The best AUC values for each sequence are listed in Table 1, showing that the best discriminative power for the prediction of surrogate subtype was found in images obtained in the mid-stage of the imaging process. Several machine learning models were trained and optimized on each MRI sequence. The best AUC values for each model are listed in Table 2, showing that several models had similar performance, with logistic regression having the best AUC over all MRI sequences.

**Table 1:** The average performance of models across different MRI sequences.

MRI Sequence	Mean ± Std AUC	MRI Sequence	Mean ± Std AUC
3_pc_sub	0.759 ± 0.043	5_pc	0.735 ± 0.027
2_pc_sub	0.758 ± 0.036	1_pc_sub	0.735 ± 0.034
3_pc	0.749 ± 0.039	feat_cov	0.730 ± 0.029
feat_concat	0.746 ± 0.048	native	0.726 ± 0.021
feat_min	0.745 ± 0.035	4_pc	0.726 ± 0.033
4_pc_sub	0.741 ± 0.038	feat_max	0.723 ± 0.020
feat_mean	0.741 ± 0.039	5_pc_sub	0.722 ± 0.029
1_pc	0.74 ± 0.034	feat_max_diff	0.718 ± 0.031
2_pc	0.737 ± 0.028	feat_std	0.699 ± 0.037

Numbering in the sequence name represents the ordering of the sequence and the suffix _sub indicates that the image is obtained as a subtraction of a post-contrast sequence with a native sequence.

**Table 2:** The performances of individual models.

Model	Mean AUC	Best AUC
Logistic regression	0.767	0.804
SVC linear	0.766	0.802
Gradient boosting	0.745	0.784
Random forest	0.732	0.774
CatBoost	0.718	0.760
SVC polynomial	0.709	0.769
SVC sigmoid	0.707	0.790

### 3.3. Model Interpretation and Feature Importances

**Figure 2:**
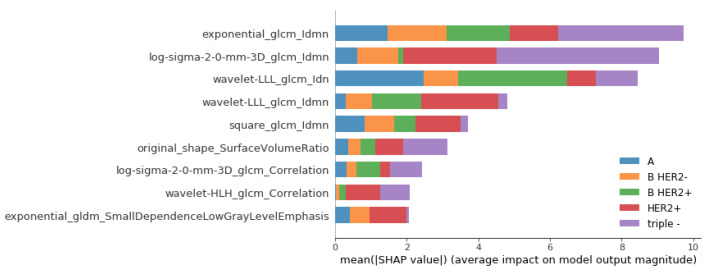
Shapley additive explanations (SHAPs) showing the contribution of different radiomic features on the output of the logistic regression model for the classification of surrogate subtypes of the breast. Larger SHAPs values indicate greater feature importance. Abbreviations for different surrogate subtypes are as follows: A—luminal A like; B HER2−—luminal B-like HER2 negative; B HER2+—luminal B-like HER2 positive; HER2+—HER2 positive; triple—triple negative.

Shapley additive explanations (SHAPs) is a method for interpreting complex machine learning models. It enables the identification of the contribution of individual features to a model’s prediction. The SHAPs method was used to explain the predictions of the logistic model that classified breast carcinomas into one of five different subtypes. The SHAPs values were computed to quantify the importance of each feature in the model, which provided insights into the association of radiomic features, preselected using the Boruta feature selection method, and surrogate subtypes. The SHAPs values were used to generate a summary plot that visualized the most important features in the classification process, shown in Figure 2. The SHAPs values, in our context, functioned as a powerful tool to elucidate the interplay between the radiomic features and the surrogate subtypes. By quantifying the importance of each feature in our model, the SHAPs values granted an understanding of how specific features influenced the model’s predictive performance and how they related to different breast carcinoma subtypes. Observing the top-most important features, it is noticeable how various pre-processing steps were useful for creation of important features, especially texture features measured by the gray-level cooccurrence matrix (GLCM), as they can capture and quantify different texture features that may not be visually perceptible but are still critical for characterizing tissues and structures in radiological images. These texture features may reflect certain pathological changes in tissues that are associated with disease progression or response to treatment, thereby serving as potential imaging biomarkers. It is also noticeable that the surface-to-volume ratio, a morphological feature, was one of the most important features. The importance of different features for each of the five surrogate subtypes is shown in Figure 2, and a detailed overview of the importance of all radiomic features is available in the Supplementary Material.

## 4. Discussion

The heterogeneity of breast cancer is one of the greatest obstacles to optimal care for breast cancer patients. A key to overcoming issues that arise from breast cancer heterogeneity is a personalized approach to each patient. The first step toward a personalized approach was the implementation of immunohistochemical biomarkers (ER, PR, HER2, and Ki-67) in the diagnostic workup of newly diagnosed breast cancer patients since they guide diagnostic and treatment decisions. However, such biomarkers can only be assessed invasively, and in the diagnostic workup of newly diagnosed breast cancer, a preferable method is a core-needle biopsy. It is well known that the most accurate identification of intrinsic breast cancer subtypes is achieved through the use of molecular techniques. However, these techniques, such as microarray-based gene expression profiling, are expensive and not widely accessible in clinical practice. To address this limitation, the 13th St. Gallen International Breast Cancer Conference Expert Panel in 2013 adopted clinicopathological surrogate definitions for the five distinct breast cancer subtypes [5]. These surrogate definitions can be routinely obtained by IHC measurements of ER, PR, Ki-67, and HER2 (with in situ hybridization confirmation, where appropriate) and are reliable enough to guide treatment decisions. Although being a recommended method, the use of core-needle biopsy for tissue analysis has some limitations, the most significant being the limited number of samples that are not representative of the entire tumor.

Diagnostic imaging through classical and deep radiomics approaches holds significant potential in preoperatively determining the breast cancer subtype. Unlike invasive biopsy, diagnostic imaging can provide a comprehensive representation of the whole tumor. However, existing radiomics studies on breast cancer subtype classification have primarily focused on distinguishing between luminal A and luminal B subtypes [16]; luminal and non-luminal subtypes [35]; HER2-positive and HER2-negative subtypes [36]; TN and non-TN subtypes [17,37]; luminal, HER2-positive, and TN subtypes [14,38], and luminal A, luminal B (it is important to note that the diagnostic criteria used for luminal B subtype are different from those in [5]), HER2-positive, and TN subtypes [39,40,41]. Additionally, several studies have been conducted to assess the expression of pathohistological prognostic factors such as ER, PR, HER2, and Ki-67 [42,43,44]. However, determining the surrogate subtype requires integrating some of these factors, which can be challenging due to the development of separate models for each task. In our study, the breast cancer subtypes were categorized based on the St. Gallen clinicopathological surrogate definitions, which carry treatment implications. Using machine-learning-based radiomics, we developed a breast cancer subtype classification model capable of classifying breast cancer into five distinct surrogate subtypes, as defined in [5]. The model achieved an overall AUC of 0.80, indicating its promising performance in accurately classifying breast cancer subtypes.

A recent systematic review and meta-analysis on the radiomic differentiation of breast cancer molecular subtypes using pre-operative breast imaging found 35 studies that evaluated breast MRI radiomics for breast cancer molecular subtype differentiation [12]. Although only studies performed by Demircioglu et al. and Choudhery et al. used subtracted images for better cancer depiction for segmentation purposes, none of these studies included both post-contrast sequences and subtracted images for radiomic analysis [45,46]. More recently, in the study conducted by You et al., the first DCE-MRI subtraction images were employed for the feature extraction and development of breast cancer subtype classification models [47]. They developed classification models that achieved the best average AUC performance for distinguishing molecular subtypes of 0.8623 [47]. Subtraction imaging is a post-processing technique that digitally subtracts a pre-contrast T1-weighted sequence from the identical sequence obtained after contrast administration. Subtraction images allow better depiction of enhancing lesions and improved detection of subtle changes in tissue vascularity and could potentially improve the accuracy of radiomic analysis for breast cancer subtype classification [48]. The results of our study suggest that radiomic features from subtracted images are important for breast cancer subtype differentiation since models that applied radiomic features extracted from the second and third post-contrast subtraction images yielded an AUC of 0.76. Although our performance metrics are slightly lower compared to the study conducted by You et al., it is important to note that there are certain differences in the dataset and methodology employed [47]. In a study conducted by You et al., they used automatic segmentation, and the breast cancers were divided into four subtypes, whereas the hormone-receptor-positive HER2-positive breast cancers were not included [47]. These discrepancies in dataset composition and subtype inclusion could potentially account for the variations observed in the performance outcomes between our study and theirs.

Unlike several other studies that only used features extracted from the first [40,46,49], second [14,35], third [17,50], and late [16] post-contrast sequence, this study included radiomic features extracted from all five post-contrast sequences of DCE-MRI of the breast since different breast cancer surrogate subtypes exhibit different angiogenic properties, which may result in different enhancement patterns during scanning and enable better surrogate subtype classification. Recently, Ming et al. revealed three novel imaging breast cancer subtypes by unsupervised learning using radiomic features extracted from multi-phase DCE-MRI [51]. The same study showed that the imaging subtypes were significantly associated with the molecular subtypes. In line with these findings, our study indicated that models incorporating radiomic features from multiple time points slightly outperformed those utilizing only radiomic features from a single-phase DCE-MRI, as evidenced by higher AUC values (0.767 as opposed to 0.759). Furthermore, our study found that models utilizing radiomic features extracted from the third post-contrast sequence achieved an AUC of 0.75, slightly outperforming the models that relied on radiomic features extracted from the first post-contrast sequence (an AUC of 0.74), which is typically regarded as the preferred source of radiomic features in most studies.

Our best models yielded better performances than models developed in one of the largest studies on molecular subtyping that included 922 patients [52]. In that study, the highest performances were obtained for the models distinguishing luminal A from other subtypes with AUC = 0.697 and triple negative from the other subtypes with AUC = 0.654, while models for determining HER2 positive vs. others and luminal B vs. others did not reach statistical significance. In our study, the highest performances were obtained for discriminating luminal A like vs. others (AUC: 0.78), luminal B-like HER2 negative vs. others (AUC: 0.57), luminal B-like HER2 positive vs. others (AUC: 0.60), HER2 positive vs. others (AUC: 0.81), and triple negative vs. others (AUC: 0.83).

This study only evaluated the performances of DCE-MRI-based radiomic features for surrogate subtype classification, while other studies included other sequences in radiomic analysis, resulting in better model performances [40,53]. However, diffusion-weighted imaging (DWI) was not used since it is known to be susceptible to artifacts and an overall lack of standardization.

When considering model and feature interpretability, it is crucial to acknowledge that classical radiomic features are generally regarded as more interpretable by radiologists compared to deep features, despite the advantages offered by deep radiomics [31]. However, the interpretability of models is often overlooked in radiomics studies focused on classifying breast cancer subtypes, which impacts their acceptance and understanding among radiologists. To address this gap, one of the primary focuses of this study was to enhance the interpretability of both the model and radiomic features by using SHAPs. Emphasizing interpretability alongside performance enhances the practical applicability and adoption of radiomics models in clinical practice and research.

By offering quantified insight into which features are driving predictions, SHAPs improves transparency and gives radiologists a greater understanding of how the model works. It also allows radiologists to critically assess the results, potentially recognizing patterns that align with their clinical knowledge or identifying areas for further investigation. This level of interpretability is particularly valuable in medical contexts, where understanding the reasoning behind a prediction is often as crucial as the prediction itself. Therefore, the inclusion of SHAPs values in our methodology serves as a significant step toward facilitating the acceptance and integration of radiomics models into the clinical workflow, by bridging the gap between machine learning outputs and clinical interpretability. The presented framework offers a comprehensive overview and a framework within which one can further investigate the utility of features, images, and models toward a better understanding of radiological images.

This study has shown that radiomic features from different categories had a different impact on the model performance and were expressed differentially for different subtypes, reflecting the underlying biological behavior among different breast cancer subtypes. These findings can be used to improve our understanding of the biological mechanisms that drive the development and progression of these tumors and thus increase the diagnostic accuracy of imaging modalities for breast cancer subtype determination to enable more effective management for specific subtypes of breast carcinoma.

Although our study resulted in several models with moderate performance, we must address this study’s limitations. The limitations of this study are the retrospective single-center nature of this study and imbalanced class data (smaller number of HER2-positive and triple-negative cancers in our cohort). However, our data reflect the distribution of surrogate subtypes in a population of patients with breast cancer. In our study, only one breast imaging expert manually segmented all breast cancers; therefore, interobserver variability was not assessed. Despite the promising results of our models, more extensive multi-center studies are required before any definitive conclusions can be drawn.

## 5. Conclusions

In conclusion, this study highlights the potential of radiomics based on features extracted from DCE-MRI for breast cancer surrogate subtype classification. We developed interpretable multi-phase DCE-MRI-based machine learning models for surrogate subtype classification. Furthermore, the developed models demonstrated an improved depiction of aggressive breast cancer subtypes, namely HER2 positive and triple negative. The reliance of the best-performing models on tissue changes occurring at the mid-term of the imaging process suggests the importance of feature extraction from multi-phase imaging. This is a novelty in the field, as the utility of subtracted images has not yet been investigated for such analyses. The accurate determination of surrogate subtypes is crucial in the diagnostic workup of breast cancer patients, as it has important implications for clinical management. The findings of this study have important clinical implications and provide a promising avenue for future research in breast cancer diagnosis and treatment.

## Data Availability

Data are contained within this article or Supplementary Material.

## Supplementary Materials

The following supporting information can be downloaded at https://www.mdpi.com/article/10.3390/jpm13071150/s1, Details on experiments and a list of radiomics features selected for each sequence can be found in the supplementary table.
